# From animal ethics to climate change: Integrating human-animal-studies and one health into medical education

**DOI:** 10.1016/j.onehlt.2026.101399

**Published:** 2026-03-30

**Authors:** K. Widmann, T.S. Busse, J. Ehlers, J. Nitsche

**Affiliations:** a Witten/Herdecke University, Department of Didactics and Educational Research in Health Science, Faculty of Health, Department for Human Medicine, Alfred-Herrhausen-Straße 50, 58455 Witten, North Rhine-Westphalia, Germany; b Witten/Herdecke University, Juniorprofessorship for Digital Health, Faculty of Health, Department for Human Medicine, Alfred-Herrhausen-Straße 50, 58455 Witten, North Rhine-Westphalia, Germany

**Keywords:** OneHealth, AnimalEthics, MedicalEducation, TransformativeLearning, SystemsThinking

## Abstract

The increasing interdependence of human, animal, and environmental health has drawn attention to educational approaches that extend beyond biomedical knowledge and engage with ethical and systemic dimensions of health within the One Health framework. This study evaluates an interdisciplinary digital lecture series on human–animal relationships developed at Witten/Herdecke University (Germany) and examines its impact on participants' ethical orientations toward animals and their understanding of One Health.

The fourteen-week lecture series, conducted during the winter semester 2024/25, combined expert-led lectures from medicine, ethics, veterinary science, law, and theology with interactive and reflective elements. A pre–post survey design was applied using the Animal Ethics Orientation Scale and adapted items from a One Health education survey. Data from students and members of the public were analysed using descriptive statistics as well as paired and independent-samples tests.

Participation in the lecture series was associated with substantial shifts toward more restrictive and animal-protective ethical attitudes. Awareness and valuation of One Health principles were also strengthened. While paired analyses showed stronger effects for animal ethics, independent-samples analyses indicated broader improvements in One Health understanding.

These findings suggest that interdisciplinary, ethics-oriented educational formats can support ethical reflection and systems thinking in health-related education. Integrating human–animal relationships into One Health teaching may contribute to the development of competencies relevant to addressing complex health challenges.

## 1 Introduction

In recent years, the accelerating loss of biodiversity, the degradation of ecosystems [1], and the continued rise of zoonotic diseases [2] have continuously highlighted the deep interconnection between human, animal and environmental health. Climate change, particularly its connection to industrial animal production, poses immediate and far-reaching health threats and its impacts are already visible within the health sector: extreme heat events and growing food insecurity increasingly strain healthcare systems, with the World Health Organization (WHO) estimating an average of 489,000 heat-related deaths annually between 2000 and 2019, disproportionately affecting vulnerable groups such as women, older adults, and individuals with chronic illnesses [3], [4]. At the same time, climate change reduces agricultural productivity, alters nutrient bioavailability, and increases pest resistance, thereby enhancing food insecurity in the long run [5]. These climate-driven pressures intersect with risks arising from modern livestock production systems. Research shows that intensive animal farming in densely populated regions contributes to respiratory health problems, zoonotic disease transmission, and the spread of antimicrobial resistance, emphasizing the broader public health burden associated with industrial livestock systems [6].

These developments increasingly influence clinical practice and public health and call for a broader understanding of the responsibilities of healthcare professionals [7]. The One Health framework recognizes the interdependence between human, animal and environmental health and has recently been defined by the One Health High-Level Expert Panel (OHHLEP) as “an integrated, unifying approach that aims to sustainably balance and optimize the health of people, animals and ecosystems” [8]. This perspective highlights that, within medical education, traditional biomedical expertise — understood as knowledge and competencies related to clinical medicine, disease diagnosis, and treatment — alone is no longer sufficient to meet the challenges of the 21st century. Rabinowitz et al. [9] argue that physicians and other health professionals should acquire an entire skill set of One Health competencies for health professionals including environmental assessment skills, understanding zoonotic diseases and animal roles, and integrating ecological and ethical considerations into medical practice. Additionally, in One Health and public health contexts, clinical practitioners are expected to complement biomedical expertise with several soft skills such as interprofessional communication, collaboration, and systems thinking, as well as personal character traits like empathy and emotional intelligence [10], [11].

Recent global health analyses highlight that climate change and ecological degradation reflect a broader “empathy gap” in the population, where the vulnerabilities of both human populations and natural systems remain underestimated [12]. At the same time, empathy has been widely recognized as a core component of high-quality patient-centred medical care [13] and is known as the most frequently mentioned personal attribute of a humanistic physician [14]. Simultaneously, healthcare education research describes empathy as not simply an innate personal attribute, but a soft skill that can be taught, learned and improved through reflective and interpersonally rich learning environments [15], [16]. This becomes even more relevant since academic literature suggests that empathy tends to decline amongst healthcare professionals during medical school and within the hospital work environment, to such an extent that it even compromises professionalism und may threaten healthcare [17]. In contrast to that, educational research argues that educational interventions that foster professional identity formation and empathy building, lead to more trusting patient-doctor-relationships, thus greater compliance, more accurate diagnosis and ultimately better patient outcomes [18], [19]. Expanding the concept of empathy beyond human relationships to include non-human animals can further enhance moral sensitivity toward vulnerable beings and improve awareness of the interconnection between human actions, animal wellbeing and environmental consciousness [20], [21].

Against this backdrop, it becomes apparent that universities, as deliverers of education to healthcare professionals and future decisionmakers, can play an important role in shaping values, attitudes, and worldviews. Higher education is increasingly recognized not only as a space for knowledge acquisition but as an environment in which learners develop ethical reasoning, civic responsibility, and critical and reflective thinking [22], [23]. The transformative learning theory provides a useful framework for understanding how such value- and behaviour-changes can occur. According to this theory, meaningful learning requires critical reflection on existing assumptions and the integration of new perspectives into one's understanding of self and world [24], [25]. Transformative learning often begins when students encounter material that is morally or emotionally challenging, encouraging them to rethink their assumptions and change their behaviour [26]. Educational formats that connect different disciplines and thus students from a broad variety of academic fields can further create a shared space for dialogue, where diverse viewpoints come together and transformative learning can take place [27]. Such approaches are closely related to the concept of interprofessional education (IPE). The World Health Organization defines IPE as situations in which individuals from two or more professions learn about, from, and with each other, with the aim of enabling effective collaboration and improving health outcomes [28]. Universities thus hold a responsibility to provide educational offers and create learning settings that invite such reflection and IPE, particularly in light of complex global health challenges.

In response to these educational needs, Witten/Herdecke University (UW/H) developed the interdisciplinary digital lecture series “Human–Animal Relationship”. Offered during the winter semester of 2024/2025, the fourteen-week series introduced participants to the ethical, ecological, medical and social dimensions of human–animal relations through weekly digital lectures delivered by experts from diverse disciplines. While the lecture series was designed to address topics relevant to health-related education, it was intentionally opened to students from different academic disciplines as well as members of the public. This approach reflects the inherently interdisciplinary nature of One Health challenges, which require collaboration across professional and societal sectors, as well as an inclusive and interdisciplinary learning environment. In addition to the lectures, the series incorporated reflective and participatory elements such as open discussions and interactive engagement tools. The first publication on this project reported on the implementation, reach, and overall acceptance of the lecture series [29]. Building upon this foundation, the present study investigates how participation in the series influenced the attitudes and values of its attendees. Specifically, the authors examine whether and to what extent engagement with the content led to shifts in participants' ethical orientations toward animals and their understanding of the interconnection between human, animal and environmental health.

### 1.1 Research questions

To evaluate the lecture series and assess its impact on participants' perspectives, attitudes, and ethical orientations toward human–animal relationships and One Health, a structured empirical study was conducted. This article will focus on the following research questions:

• To what extent do participants, prior to the lecture series, perceive human–animal relationships and One Health as relevant to human, animal, and planetary health?
• How do participants' perspectives change during and after participation in the lecture series “Human–Animal Relationship” in the winter semester 2024/2025?

Building on the previously published description of the lecture series' implementation [29], the present study focuses on the empirical evaluation of its educational impact.

## 2 Materials and methods

Ethical approval for this study was granted by the Ethics Committee of UW/H (reference number 186/2024).

### 2.1 Intervention

**Fig. 1:**
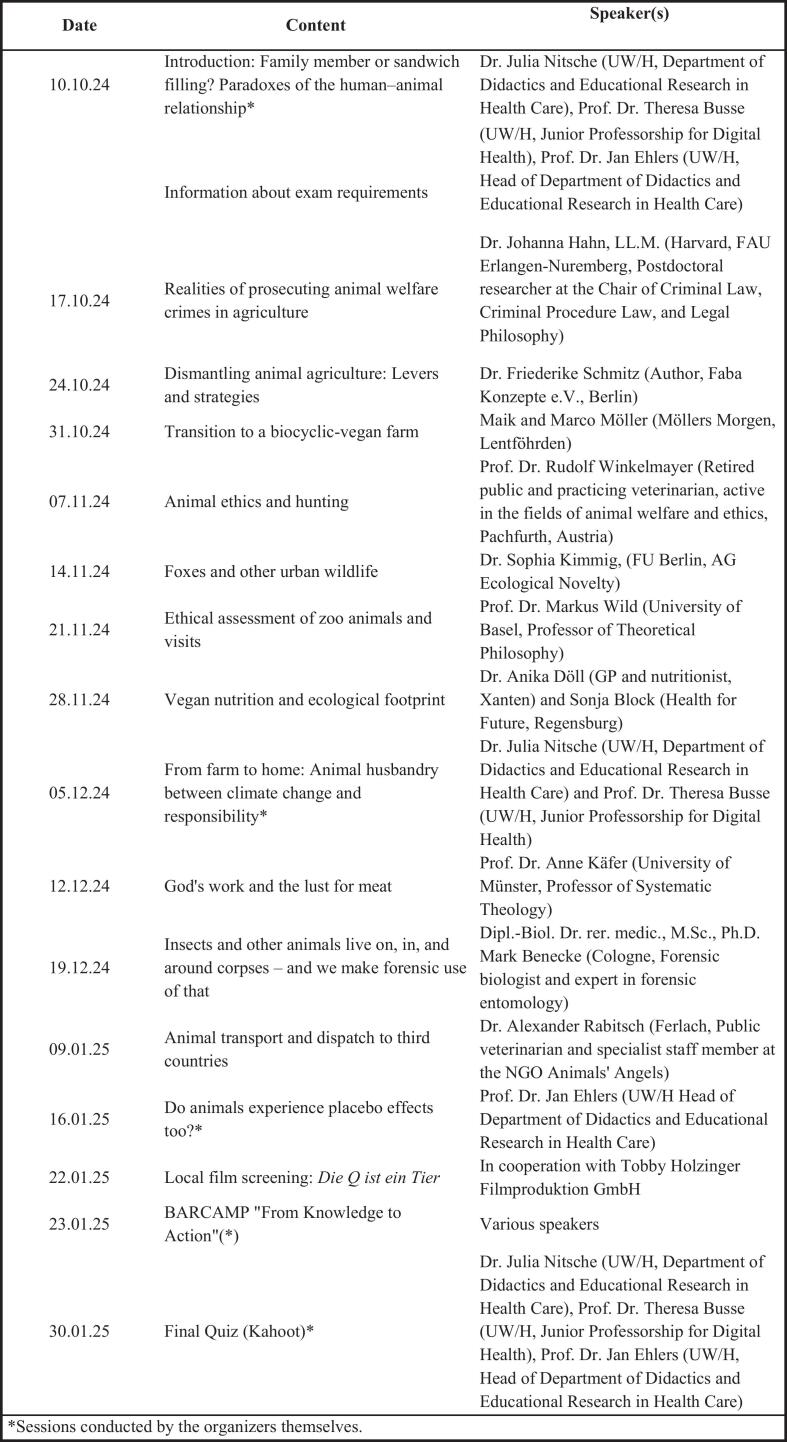
Contents of the lecture series in the winter semester of 2024/2025.

Designed as a 14-week interdisciplinary course, the lecture series “Human-Animal Relationship” was offered during the winter semester 2024/2025 as part of the university's *Studium Fundamentale* program and was open to both students and the public. Each 90-min session featured impulse lectures from invited experts from fields such as medicine, veterinary science, ethics, law, and theology, addressing diverse dimensions of human–animal relations and One Health and was followed by a subsequent open discussion. The course lecturers (J.N., J.E., T.S.B.) moderated the discussions and delivered some of the impulse lectures (see Fig. 1). The lecture series incorporated interactive components including live polls and Q&A discussions, which encouraged collaborative reflection and the transfer of learning into practice. Additionally, a participatory *Barcamp* workshop, a participant-driven, collaboratively organized “unconference” where attendees co-create the agenda and engage in small-group sessions on self-selected topics [30] titled *“From Knowledge to Action”* was conducted.

A public film screening and a final interactive quiz session complemented the program, fostering emotional engagement and reinforcing learning outcomes. Conducted online via the platform Zoom (Zoom Video Communications, Inc., 55 Almaden Blvd, 6th Floor, San Jose, CA 95113, USA) and supplemented by publicly available YouTube recordings of the sessions (made available after approval by the respective speakers), the format enabled wide accessibility and interdisciplinary participation.

### 2.2 Participants

The lecture series was open to both internal students of UW/H and external participants, including students from other universities and interested members of the public. A substantial proportion of participants were students enrolled in health-related programs such as human medicine, dentistry, and psychology. Participation in the study was restricted to individuals aged 18 years or older who attended the majority of sessions (at least 12 out of 16; self-disclosure). All data was pseudonymized. Participants generated an individual code to link pre- and post-survey responses without revealing their identity. Participation was voluntary and based on informed consent in accordance with the General Data Protection Regulation (GDPR).

### 2.3 Data collection

To answer the research questions, a pseudonymized, non-experimental cross-sectional survey (pre–post design) was conducted. Participants completed a digital questionnaire via the online survey tool Limesurvey (LimeSurvey GmbH, Papenreye 63, 22453 Hamburg, Germany) at two points in time. The pre-survey questionnaire was introduced at the beginning of the lecture series during the first and second session (T1), which took place on the 10th of October 2024 and 17th of October 2024 respectively. The post-survey questionnaire was distributed at the end of the lecture series during the penultimate and final sessions (T2) on the 23rd of January 2025 and 30th of January 2025 respectively. This time frame had been chosen to ensure the highest possible response rate as well as the most comprehensive impression of the participants on the lecture series and also in advance. Each survey required approximately 20 min to complete. Participants accessed the questionnaire through personalized course communication channels (Zoom chats, Moodle and mailing lists).

### 2.4 Survey design

At the beginning of the questionnaire, participants were asked to create a pseudonym. This pseudonym consisted of the first letter of their mother's first name (one digit), the first letter of their place of birth (one digit), the last number of their current ZIP code (one digit) and the first two numbers of their date of birth (two digits). This procedure allowed participants to withdraw their data after completing the survey by providing their pseudonym (anonymously if they wished) and enabled the researchers to match data from the pre- and post-surveys.

The questionnaire (Appendix A) consisted of four main sections addressing both sociodemographic and attitudinal aspects. The first section (Section A) collected background information such as gender, age, educational level, field of study, university affiliation, and professional or voluntary engagement in health-related activities, as well as personal involvement in climate or animal protection and pet or livestock ownership. The second section (Section B) measured participants' ethical perspectives on the human use of animals using the *Animal Ethics Orientation Scale* [31] comprising 13 items on a six-point Likert scale ranging from 1 = *strongly disagree* to 6 = *strongly agree*. The third section (Section C) assessed participants' views on the interconnection between human, animal, and environmental health through nine items adapted on a six-point Likert scale from the *K–12 One Health Education Survey* [32] which also addressed ecological sustainability and ethical responsibility. The final section (Section D), included only in the post-survey, asked participants to evaluate the relevance of these topics for higher education curricula and to provide qualitative feedback and suggestions for curricular integration.

The translation of Sections B and C was conducted independently by two individuals. First, Person 1 (J.N.) translated the scales into German. Person 2 (P.T.), unfamiliar with the original version, back-translated them into English. Both then compared the back-translation for inconsistencies and jointly finalized the German versions.

When opening the online questionnaire, participants were informed about the objectives of the study, data protection procedures in accordance with Art. 6 (1)(a) GDPR, and the voluntary nature of their participation. Participants were also informed of their right to withdraw from the study at any time without providing a reason. Contact information for the Data Protection Officer of UW/H was provided, and participants had the opportunity to ask questions prior to participation. Only after confirming informed consent were participants granted access to the questionnaire.

### 2.5 Data analysis

All analyses were conducted using SPSS (Version 30.0, IBM Corporation, 233 S. Wacker Drive, 11th Floor, Chicago, IL 60606–6307, USA). Descriptive statistics were calculated for all items of the Animal Ethics Orientation and One Health scales at baseline (T1) and post-intervention (T2). To assess within-person changes amongst participants who completed both surveys, paired-samples *t*-tests were performed for each item. Additionally, Wilcoxon signed-rank tests were additionally conducted as non-parametric counterparts to the paired *t*-tests. Assumptions of normality for paired differences were examined using Shapiro–Wilk tests.

To complement the paired analyses, independent-samples t-tests were conducted comparing all T1 and T2 respondents. For each item, Levene's test was used to assess homogeneity of variance and determine whether the “equal variances assumed” or “not assumed” estimate should be interpreted. Effect sizes for parametric tests were expressed as Cohen's d. Statistical significance was defined as *p* < .05 (two-tailed).

In addition to the quantitative items, the questionnaire included an optional free-text response section (Section D). The research team reviewed these responses to gain general insights into participants' impressions of the lecture series. However, as these free-text responses do not address the predefined research questions the results are not to be presented here.

## 3 Results

A total of 1382 individuals registered for the Human–Animal Relationship lecture series during the winter semester 2024/2025. The mean number of participants per session was 470.5 (*SD* = 60.9).

Overall, 322 participants participated in the pre-survey (T1) and 281 in the post-survey (T2). After data cleaning and the removal of duplicates and incomplete responses (dropouts on page 5 or before), 395 valid questionnaires across both time points (260 for T1 and 159 for T2) were retained for analysis.

At baseline (T1), the sample consisted predominantly of women (*n* = 188; 72.3%), followed by men (*n* = 68; 26.2%) and individuals identifying as non-binary or gender-queer (*n* = 3; 1.2%). One participant (0.4%) did not report gender. A majority indicated that they were currently enrolled in university studies (*n* = 214; 82.6%), while 45 (17.4%) were not. Concerning institutional affiliation, 203 participants reported studying at UW/H (94.9%), with 11 (5.1%) participants listing other universities. The 214 students represented a range of academic disciplines: Human Medicine (*n* = 105; 49.1%), Dental Medicine (*n* = 55; 25.7%), Psychology (*n* = 38; 17.8%), Management (n = 5; 2.3%) and several individual programs grouped under “Other” (*n* = 11; 5.1%). 34.6% (*n* = 90) of the participants indicated current employment in health-related settings, whereas 26.9% (*n* = 70) stated having worked in the health sector in the past. 46.9% (*n* = 122) stated that they were currently pet owners and 2.7% (*n* = 7) indicated being livestock owners, with 5.8% (*n* = 15) revealing they had owned livestock in the past.

At follow-up (T2), the gender distribution remained similar, with most respondents identifying as female (*n* = 119; 74.8%), followed by male (*n* = 39; 24.5%) and one non-binary/gender-queer participant (0.6%). 55.3% indicated they were currently studying (*n* = 88), with 88.6% of the students being enrolled at UW/H (*n* = 78) and 11.4% stated being enrolled at other universities (*n* = 10). 56.8% were studying Human Medicine (*n* = 50), 19.3% Dental Medicine (*n* = 17), 11.4% Psychology (*n* = 10), 1.1% Management *(n* = 1) and 11.4% from several other degree programs (*n* = 11). 28.9% indicated working in the health sector (*n* = 46), whereas 27% revealed having worked there previously (*n* = 43). Amongst the participants at T2 48.4% were current pet owners (*n* = 77) and 48.4% as well indicated having owned pets in the past (*n* = 77). Only four participants (2.5%) indicated being livestock owners at the moment, however 13 (8.2%) people stated having owned livestock in the past.

### 3.1 Animal ethics orientation (T1-T2)

Descriptive results indicated substantial mean shifts across almost all items of the Animal Ethics Orientation Scale (Table 1). At baseline (T1), participants expressed moderate agreement with items reflecting concern for animal welfare (e.g., *“The use of animals by humans should be prohibited by law”*; *M* = 3.20) and comparatively lower agreement with items permitting or justifying the use of animals (e.g., *“We have the right to use animals because humans are intellectually superior”*; *M* = 1.75).

**Table 1:** Mean values for the Animal Ethics Orientation Survey Items at T1 and T2 (6-point Likert scale: 1 = strongly disagree; 6 = strongly agree).

Item	Mean T1 (Two decimal places)	Mean T2 (Two decimal places)
1. The use of animals by humans should be prohibited by law.	3.20 (SD = 1.61, *n* = 254)	4.26 (SD = 1.67, *n* = 159)
2. In principle, the use of animals by humans is unacceptable because animals can feel pain, happiness, etc.	3.83 (SD = 1.68, *n* = 253)	4.73 (SD = 1.53, *n* = 158)
3. In principle, the use of animals by humans is unacceptable because animals are sentient beings.	3.95 (SD = 1.64, *n* = 252)	4.76 (SD = 1.54, *n* = 158)
4. We have the right to use animals because humans are intellectually superior to animals.	1.75 (SD = 1.11, *n* = 254	1.47 (SD = 1.12, *n* = 158)
5. Human interests are more important than those of animals.	2.22 (SD = 1.35, *n* = 255)	1.92 (SD = 1.32, n = 158)
6. We must prioritize humans over animals.	2.59 (SD = 1.50, *n* = 253)	2.03 (SD = 1.31, n = 158)
7. It is acceptable for humans to put animals down if it is done painlessly.	4.33 (SD = 1.40, *n* = 251)	4.04 (SD = 1.51, *n* = 156)
8. Using animals for important human purposes (e.g. medical research) is acceptable if it is done so that the animals do not experience unnecessary stress.	3.66 (SD = 1.67, *n* = 255)	2.85 (SD = 1.59, *n* = 158)
9. Using animals for important human purposes is acceptable if it is done so that the animals do not experience unnecessary pain.	3.69 (SD = 1.58, n = 255)	2.76 (SD = 1.58, *n* = 158)
10. Using animals for important human purposes is acceptable if the animals have a decent quality of life.	3.80 (SD = 1.58, *n* = 256)	2.90 (SD = 1.59, *n* = 158)
11. Inflicting serious pain on animals is acceptable if it is necessary in order to achieve a vital human goal–e.g. in medical research.	1.86 (SD = 1.26, n = 256)	1.51 (SD = 1.11, n = 158)
12. Inflicting considerable pain on animals is justified if the purpose is sufficiently important—e.g. medical research.	1.84 (SD = 1.27, *n* = 255)	1.55 (SD = 1.10, *n* = 157)
13. Exposing animals to stress and reducing their welfare is justified if the purpose is sufficiently important.	2.00 (SD = 1.26, *n* = 254)	1.70 (SD = 1.07, *n* = 158)

At T2, after participating in the lecture series, means consistently shifted in the expected direction: participants reported higher agreement with animal-protective statements (e.g., item 1: *M* = 4.26) and lower agreement with anthropocentric or harm-justifying statements (e.g., item 6: *M* = 2.03; item 11: *M* = 1.51). These descriptive patterns already suggested a broad shift toward more critical and ethically restrictive attitudes toward human uses of animals.

### 3.2 One health

Descriptive results for the One Health items (Table 2) indicated generally high baseline endorsement at T1, reflecting a pre-existing awareness of the interconnectedness of human, animal, and environmental health amongst participants (e.g., *“The health of humans, other animal species and plants cannot be separated”; M* = 4.00). Nevertheless, means increased further across all nine items at T2, suggesting that the lecture series strengthened participants' understanding and valuation of One Health principles despite already elevated initial ratings.

**Table 2:** Mean values for the One Health Education Survey Items at T1 and T2 (6-point Likert scale: 1 = strongly disagree; 6 = strongly agree).

Item	Mean T1 (two decimal places)	Mean T2 (two decimal places)
1. The health of humans, other animal species and plants cannot be separated.	4.00 (SD = 1.68, *n* = 239)	4.62 (SD = 1.66, *n* = 151)
2. “Environment” includes natural and built environments.	4.50 (SD = 1.36, *n* = 241)	4.84 (SD = 1.47, *n* = 148)
3. Humans have a moral imperative to address One Health challenges.	4.95 (SD = 1.19, *n* = 234)	5.22 (SD = 1.25, n = 148)
4. One Health should be practiced so that there is no net (ecosystem) loss of biological diversity.	4.98 (SD = 1.21, *n* = 232)	5.24 (SD = 1.17, *n* = 147)
5. One Health embraces the value of social interaction as a critical component of health and well-being.	4.67 (SD = 1.16, *n* = 218)	5.04 (SD = 1.22, *n* = 145)
6. The World Health Organization defines “health” as “a state of complete physical, mental and social well-being and not merely the absence of disease or infirmity.” This definition also applies to other animals and ecosystems.	4.88 (SD = 1.17, *n* = 232)	5.33 (SD = 1.10, *n* = 150)
7. One Health recognizes the intrinsic value of life on earth (plants, animals, microbes) regardless of a direct benefit to humans.	4.78 (SD = 1.14, *n* = 214)	5.15 (SD = 1.25, *n* = 144)
8. Ecological, economic, social/cultural, ethical and justicial sustainability are equally important for One Health.	4.51 (SD = 1.29, *n* = 213)	4.97 (SD = 1.21, n = 144)
9. When you optimize health for one species, health for others is marginalized or eliminated.	3.26 (SD = 1.42, *n* = 222)	3.61 (SD = 1.67, *n* = 145)

### 3.3 Effect of the lecture series on participant's attitudes

In order to evaluate pre–post changes in participant's attitudes and mindsets, 45 pseudonymized pairs at T1 and T2 were extracted from the datasets for statistical testing.

Shapiro–Wilk tests indicated that most item difference scores deviated from normality. However, considering the sample size (*n* = 45) and robustness of the *t*-test, both paired *t*-tests and additional Wilcoxon signed-rank tests were conducted. Both results are reported in Table 3.

**Table 3:** Results of paired samples *t*-test and Wilcoxon signed rank test on pseudonymized pairs.

Item	t-Wert	df (t)	p (t-Test)	Wilcoxon Z	p (Wilcoxon)	Cohen's d (t-Test)
1. The use of animals by humans should be prohibited by law.	−4.237	44	< 0.001	−3.505	< 0.001	−0.632
2. In principle, the use of animals by humans is unacceptable because animals can feel pain, happiness, etc.	−2.284	44	0.027	−2.459	0.014	−0.341
3. In principle, the use of animals by humans is unacceptable because animals are sentient beings.	−2.007	44	0.051	−2.090	0.037	−0.299
4. We have the right to use animals because humans are intellectually superior to animals.	2.199	44	0.033	−2.292	0.022	0.328
5. Human interests are more important than those of animals.	0.666	44	0.509	−0.776	0.438	0.099
6. We must prioritize humans over animals.	1.935	44	0.059	−1.840	0.066	0.288
7. It is acceptable for humans to put animals down if it is done painlessly.	2.296	44	0.027	−2.205	0.027	0.342
8. Using animals for important human purposes (e.g. medical research) is acceptable if it is done so that the animals do not experience unnecessary stress.	3.312	44	0.002	−3.078	0.002	0.494
9. Using animals for important human purposes is acceptable if it is done so that the animals do not experience unnecessary pain.	3.313	44	0.002	2932	0.003	0.494
10. Using animals for important human purposes is acceptable if the animals have a decent quality of life.	1.132	44	0.264	−1.228	0.219	0.169
11. Inflicting serious pain on animals is acceptable if it is necessary in order to achieve a vital human goal–e.g. in medical research.	1.762	44	0.085	−1.577	0.115	0.263
12. Inflicting considerable pain on animals is justified if the purpose is sufficiently important—e.g. medical research.	2.331	44	0.024	−2.218	0.027	0.347
13. Exposing animals to stress and reducing their welfare is justified if the purpose is sufficiently important.	2.602	44	0.013	−2.410	0.016	0.388
OH1. The health of humans, other animal species and plants cannot be separated.	−1.504	41	0.140	−1.643	0.100	−0.232
OH2. “Environment” includes natural and built environments.	−1.987	41	0.054	−1.890	0.059	−0.307
OH3. Humans have a moral imperative to address One Health challenges.	−0.158	41	0.875	−0.214	0.831	−0.024
OH4. One Health should be practiced so that there is no net (ecosystem) loss of biological diversity.	−1.639	39	0.109	−1.615	0.106	−0.259
OH5. One Health embraces the value of social interaction as a critical component of health and well-being.	−2.737	37	0.009	−2.607	0.009	−0.444
OH6. The World Health Organization defines “health” as “a state of complete physical, mental and social well-being and not merely the absence of disease or infirmity.” This definition also applies to other animals and ecosystems.	−0.489	41	0.628	−0.774	0.439	−0.075
OH7. One Health recognizes the intrinsic value of life on earth (plants, animals, microbes) regardless of a direct benefit to humans.	−0.598	38	0.553	−1.131	0.258	−0.096
OH8. Ecological, economic, social/cultural, ethical and justicial sustainability are equally important for One Health.	−0.879	37	0.385	−0.701	0.483	−0.143
OH9. When you optimize health for one species, health for others is marginalized or eliminated.	−1.331	40	0.191	−1.224	0.221	−0.208

Paired t-tests and Wilcoxon signed-rank tests (Table 3) revealed consistent within-person changes aligned with descriptive trends. Significant increases were observed in restrictive attitudes toward animal use, including greater endorsement of prohibiting or morally rejecting human use of animals. For example, agreement with the statement *“The use of animals by humans should be prohibited by law”, “In principle, the use of animals by humans is unacceptable because animals can feel pain, happiness, etc.”* or *“In principle, the use of animals by humans is unacceptable because animals are sentient beings”* significantly increased.

Participants also showed decreases in acceptance of conditional justifications for animal use, such as statements framing use as acceptable in the absence of unnecessary stress or pain or for medical research purposes. For instance agreement with the statements “*Using animals for important human purposes (e.g. medical research) is acceptable if it is done so that the animals do not experience unnecessary stress”*, “*Using animals for important human purposes is acceptable if it is done so that the animals do not experience unnecessary pain”* or *“Inflicting considerable pain on animals is justified if the purpose is sufficiently important—e.g. medical research”* significantly decreased. Anthropocentric attitudes, including claims of intellectual superiority as a justification for animal use, also declined significantly. Overall, 9 out of the 13 question items from the animal ethics questionnaire showed a significant change in the participant's perception.

Amongst the One Health items, only the statement *“One Health embraces the value of social interaction as a critical component of health and well-being”* reached significance in the paired analysis. Although other One Health items trended in the expected direction in the descriptive analysis, there was no statistical significance in the paired analysis.

Independent samples t-tests comparing all pre-intervention (T1) with all post-intervention (T2) respondents (Table 4) replicated and strengthened the previous findings. Levene's test was used to determine whether equal variances could be assumed for each item. With the exception of Item 7 (*“It is acceptable to euthanize animals when it can be done painlessly”*), all Animal Ethics items showed statistically significant differences, with effect sizes ranging from small to large (e.g., Item 1: *d* ≈ 0.65; Item 9: *d* ≈ 0.59).

**Table 4:** Results of independent samples *t*-test on pseudonymized pairs.

Item	Sig. (Levene's. Test for Equality of Variances)	t	df	p (two-sided); t-test for Equality of Means)	Cohen's d
1. The use of animals by humans should be prohibited by law.	0.718	−6.429	411	< 0.001	−0.65
2. In principle, the use of animals by humans is unacceptable because animals can feel pain, happiness, etc.	0.014	−5.556	409	< 0.001	−0.55
3. In principle, the use of animals by humans is unacceptable because animals are sentient beings.	0.051	−4.957	408	< 0.001	−0.50
4. We have the right to use animals because humans are intellectually superior to animals.	0.048	2.451	330.388	0.015	0.25
5. Human interests are more important than those of animals.	0.033	2.190	337.510	0.029	0.22
6. We must prioritize humans over animals.	<0.001	4.022	366.235	<0 0.001	0.40
7. It is acceptable for humans to put animals down if it is done painlessly.	0.284	1.915	405	0.056	0.20
8. Using animals for important human purposes (e.g. medical research) is acceptable if it is done so that the animals do not experience unnecessary stress.	0.337	4.902	411	< 0.001	0.40
9. Using animals for important human purposes is acceptable if it is done so that the animals do not experience unnecessary pain.	0.849	5.806	411	< 0.001	0.59
10. Using animals for important human purposes is acceptable if the animals have a decent quality of life.	0.681	5.641	412	<0 0.001	0.57
11. Inflicting serious pain on animals is acceptable if it is necessary in order to achieve a vital human goal–e.g. in medical research.	0.033	2.951	363.852	0.003	0.29
12. Inflicting considerable pain on animals is justified if the purpose is sufficiently important—e.g. medical research.	0.020	2.456	366.492	0.015	0.24
13. Exposing animals to stress and reducing their welfare is justified if the purpose is sufficiently important.	0.102	2.466	410	0.14	0.25
OH1. The health of humans, other animal species and plants cannot be separated.	0.811	−3.548	388	<0.001	−0.37
OH2. “Environment” includes natural and built environments.	0.653	−2.288	387	0.023	−0.24
OH3. Humans have a moral imperative to address One Health challenges.	0.831	−2.097	380	0.37	−0.22
OH4. One Health should be practiced so that there is no net (ecosystem) loss of biological diversity.	0.261	−2.055	377	0.041	−0.22
OH5. One Health embraces the value of social interaction as a critical component of health and well-being.	0.174	−2.971	361	0.003	−0.32
OH6. The World Health Organization defines “health” as “a state of complete physical, mental and social well-being and not merely the absence of disease or infirmity.” This definition also applies to other animals and ecosystems.	0.197	−3.691	380	<0.001	−0.39
OH7. One Health recognizes the intrinsic value of life on earth (plants, animals, microbes) regardless of a direct benefit to humans.	0.969	−2.900	356	0.004	−0.31
OH8. Ecological, economic, social/cultural, ethical and justicial sustainability are equally important for One Health.	0.044	−3.470	319.160	<0.001	−0.37
OH9. When you optimize health for one species, health for others is marginalized or eliminated.	0.006	−2.096	271.656	0.037	−0.23

Importantly, all nine One Health items were significant in the independent-samples analysis. Although effect sizes were generally small to moderate (d ≈ 0.20–0.40), the pattern indicates widespread enhancement of One Health understanding amongst the participants.

## 4 Discussion

The aim of this study was to investigate whether participation in an interdisciplinary lecture series addressing human–animal relationships was associated with changes in participants' perspectives, attitudes, and ethical orientations toward human–animal relationships and One Health. The fourteen-week-long lecture series featured weekly expert-led lectures, was conducted digitally via Zoom and was open to both students and the general public. At the beginning (T1) and at the end (T2) of the lecture series, participants were asked to fill out a questionnaire in order to assess the impact of the educational intervention. The results show substantial shifts in animal-ethics orientations, whereas changes in One Health attitudes were scarcer in paired analyses but apparent in independent samples comparisons.

From a behavioural psychology perspective, several mechanisms may explain why these changes occurred amongst participants. A review by Dolcos et al. [33] suggested strong evidence for the notion that emotion, and thus emotionally charged content, enhances long-term episodic memory and attention amongst learners. Immordino-Young and Damasio [34] further build on this by arguing that the connection between emotion and cognition, which takes place every time individuals recognize and respond to external influences, drives the human ability for morality and ethical thought. In addition to this, ethics-focused education has been shown to promote moral reasoning and value building, especially when learners are confronted with ethical dilemmas and are required to not only find arguments for their own position but have to engage with and gather in other opinions too [35]. Particularly in a medical education context, educational formats such as moral case deliberation, ethics rounds, or discussion groups can enhance learners' ethical reflection on certain topics [36]. On top of this, topics related to animal welfare and suffering often evoke strong emotional responses [21] that may strengthen reflectional processes and may have further contributed to the strong attitudinal changes observed in the animal ethics domain of the survey.

The lecture series may have also supported the development of systemic thinking by encouraging participants to engage with the interconnectedness between animal welfare, environmental degradation, and public and human health. Understanding these interactions is a core element of One Health and systems thinking, which encompasses competencies such as recognizing interconnections, identifying and understanding feedback, non-linear relationships and dynamic behaviour and understanding system structures [37]. However, research suggests that systems-oriented learning is an iterative process that goes through several stages, which may take a longer period of time [38]. The comparatively smaller changes observed in the paired analyses of the One Health survey items may therefore reflect the complexity of these concepts and the limited duration of the lecture series rather than a lack of educational impact. The significant differences in the independent samples analyses across all One Health survey items suggest that the intervention nevertheless contributed to strengthening participants' awareness and knowledge about One Health – a short-term outcome that has been reported by previous studies focusing on educational One Health interventions [39].

In summary, the observed attitudinal changes may reflect learning processes across multiple dimensions. On a cognitive level, participants likely acquired knowledge about animal ethics and One Health concepts and deepened their systems-oriented way of thinking about the interconnectedness of these issues. On an affective level, engagement with emotionally charged topics, such as animal welfare, may have improved the participants' attention and memorization of the contents [40], which in turn may have increased empathic sensitivity and moral awareness. And lastly on a conative level, these attitudinal changes may have influenced participants' readiness to act in more morally and ethically responsible ways, even though behavioural outcomes were not measured directly. Educational research suggests that by combining emotion [41] together with motivation and cognition, rather than just isolated knowledge acquisition, transformative learning outcomes can be much more impactful and long-term.

Another important factor contributing to the observed attitudinal changes may have been the interdisciplinary character of the lecture series. Interdisciplinary settings enable learners to encounter different knowledge cultures, norms and values [42], which can stimulate reflection and integrative thinking [43]. Since this interdisciplinarity was represented not only in the multifaceted content of the lecture series – ranging from ethics to medicine and veterinary science to environmental studies - and the lecturers presenting the topics, but also within the participant's educational backgrounds, the lecture series may have contibuted to questioning established assumptions and opinions. Particularly in a One Health context, academic literature suggests that this interdisciplinarity is not only essential in learning environments [44] but for tackling One Health issues as a whole. Bammer [45] expands on this view by stating that through interdisciplinary approaches each discipline can contribute in-depth understanding of specific topics and issues, while simultaneously also overcoming its own limitations and broadening their view in order to tackle the inherent complexity of nature and society.

## 5 Conclusions

These findings have several implications for future education and practice. Through the lecture series, students in health-related professions were able to improve their moral reasoning, ethical decision-making, and systems thinking skills – competencies, that are widely recognized as core soft skills for One Health practitioners [10]. By learning about complex, values-based challenges, students were asked to reflect on ethical and moral dilemmas across human, animal and environmental domains. In this way, universities can contribute not only to knowledge acquisition, but also to students' personal development and their capacity to address ethical challenges in a professional context. At the same time, such education formats may generate broader societal benefits by addressing existing gaps in One Health education. Despite growing advocacy for including One Health in medical curricula, there remains a scarcity of tangible One Health curricular materials, practical course contents and implementation strategies [46]. This project seeks to contribute to closing this gap by developing and evaluating an integrative, interdisciplinary education format for future health professionals.

Overall, this study suggests that interdisciplinary, ethics-oriented educational formats can meaningfully influence ethical attitudes and enhance deeper reflection on the interconnection between animals, humans and the environment. The findings indicate that such learning environments play an important role in shaping moral sensitivity, empathy, and systems awareness. Integrating these approaches more systematically and longitudinally within healthcare curricula may help prepare future professionals for the ethical and ecological challenges of the 21st century.

### 5.1 Limitations

Several limitations must be considered. The voluntary nature of the lecture series may have resulted in self-selection bias, since individuals who were already interested in these topics could have been more likely to participate. Furthermore, attitudinal changes may have been influenced by factors beyond the intervention itself, such as prior courses, informal discussions with peers, or parallel learning experiences that could not be controlled and were not queried. It also remains questionable, to what extent the attitudinal changes actually translate to behavioural changes amongst the participants outside of the lecture series. Lastly, it should be noted that due to the pseudonymization process, some participants could not be matched across both measurement points. Consequently, the availability of paired data was reduced and should be interpreted with caution. Nevertheless, the findings suggest that even within these constraints, interdisciplinary, values-based educational formats can contribute to shaping attitudes, empathy, and systemic awareness.

## CRediT authorship contribution statement

**K. Widmann:** Writing – original draft, Visualization, Formal analysis, Data curation. **T.S. Busse:** Writing – review & editing, Validation, Supervision, Methodology, Investigation, Conceptualization. **J. Ehlers:** Writing – review & editing, Supervision, Resources, Conceptualization. **J. Nitsche:** Writing – review & editing, Validation, Supervision, Project administration, Methodology, Investigation, Conceptualization.

## Informed consent statement

Informed consent was obtained electronically from all participants prior to their participation in the study.

## Institutional review board statement

Ethical approval for this study was granted by the ethics committee of Witten/Herdecke University (reference number 186/2024).

## Funding

This research received no external funding.

## Declaration of competing interest

The authors declare that they have no known competing financial interests or personal relationships that could have appeared to influence the work reported in this paper.

## Figures and Tables

**Table:** 

Teil	Frage	Antwortmöglichkeiten
**Teil A: Demographisches**	Bitte geben Sie hier Ihr biologisches Geschlecht an	• Weiblich • Männlich • nicht-binär / gender-queer • kein Geschlecht • keine Angabe
	Bitte geben Sie hier Ihr Alter an (Alter in Jahren, bspw. 30)	*_____* Jahre
	Mit wie vielen Personen wohnen Sie in einem Haushalt? (1 = ich wohne alleine)	_____ (numerische Eingabe)
**Teil A: Hochschul-Bildung**	Was ist Ihr höchster allgemeinbildender Schulabschluss?	• Ich bin von der Schule abgegangen ohne Hauptschulabschluss (Volksschulabschluss) • Ich habe den Hauptschulabschluss (Volksschulabschluss) • Ich habe den Realschulabschluss (Mittlere Reife) • Ich habe den Abschluss der Polytechnischen Oberschule 10. Klasse (vor 1965: 8. Klasse) • Ich habe die Fachhochschulreife • Ich habe die allgemeine/fachgebundene Hochschulreife/Abitur • Ich habe einen anderen Schulabschluss, und zwar: __________________________
	Ich studiere an folgender Hochschule:	• Universität Witten/Herdecke • Andere: _______________
	Ich studiere im folgenden Studiengang:	• Humanmedizin • Zahnmedizin • Philosophie, Politik und Ökonomik (B.A.) • Management (B.Sc.) • General Management (M.A.) • Psychologie/Psychotherapie (B.Sc.) • Klinische Psychologie/Psychotherapie (M.Sc.) • Community Health Nursing (M.Sc.) • Ethik und Organization (M.A.) • Pflegewissenschaft (M.Sc.) • Andere: _____________________
	Der Abschluss meines aktuellen Studiengangs ist:	• Bachelor () • Master () • Staatsexamen () • Andere: _________
	Ich bin in folgendem Semester	_____ (numerische Eingabe)
**Teil A:** **Sonstiges**	Bezug zur Gesundheitsversorgung:	• Ich arbeite selbst im Gesundheitsbereich. o wenn ja: Seit wie vielen Jahren? o wenn ja, in folgendem Beruf: _______ • Ich habe selbst im Gesundheitsbereich gearbeitet, jetzt jedoch nicht mehr. o wenn ja: Wie viele Jahre? o wenn ja, in folgendem Beruf: ________
	Engagement:	• Ich engagiere mich aktiv für den Tierschutz. (1 = stimme gar nicht zu, 6 = stimme völlig zu) • Ich engagiere mich aktiv für den Klimaschutz, z. B. in einer Campus for Future Aktivist:innen Gruppe. (1 = stimme gar nicht zu, 6 = stimme völlig zu)
	Haustiere	• Ich habe selbst aktuell Haustiere (Ja / Nein) • Ich hatte selbst schon einmal Haustiere, jetzt aktuell jedoch nicht mehr (Ja / Nein) • Wenn ja, wieviele Haustiere? • Wenn ja, welche Haustiere?
	Nutztiere	• Ich habe selbst aktuell Nutztiere (Ja / Nein) • Ich hatte selbst schon einmal Nutztiere, jetzt aktuell jedoch nicht mehr (Ja / Nein) • Wenn ja, wieviele Nutztiere? • Wenn ja, welche Nutztiere?
**Teil B**	**Animal Ethics Orientation (Lund et al., 2019)** Nachfolgend finden Sie einige Aussagen zur Beziehung zwischen Mensch und Tier. Geben Sie bitte für jede Aussage an, in welchem Maße Sie mit ihr übereinstimmen. (1 = stimme gar nicht zu, 6 = stimme völlig zu)	1. Die Verwendung von Tieren durch den Menschen sollte gesetzlich verboten werden. 2. Grundsätzlich ist die Nutzung von Tieren durch den Menschen inakzeptabel, da Tiere Schmerz, Glück usw. Empfinden können. 3. Grundsätzlich ist die Nutzung von Tieren durch den Menschen nicht akzeptabel, weil Tiere fühlende Wesen sind. 4. Wir haben das Recht, Tiere zu nutzen, weil der Mensch dem Tier intellektuell überlegen ist. 5. Die Interessen des Menschen sind wichtiger als die der Tiere. 6. Wir müssen dem Menschen Vorrang vor dem Tier geben. 7. Es ist für Menschen akzeptabel, Tiere einzuschläfern, wenn dies ohne Schmerzen geschieht. 8. Die Verwendung von Tieren für wichtige menschliche Zwecke (z. B. medizinische Forschung) ist akzeptabel, wenn dies so geschieht, dass die Tiere nicht unnötig gestresst werden. 9. Die Verwendung von Tieren für wichtige menschliche Zwecke ist akzeptabel, wenn sie so erfolgt, dass die Tiere nicht unnötige Schmerzen erleiden. 10. Die Verwendung von Tieren für wichtige menschliche Zwecke ist akzeptabel, wenn die Tiere eine angemessene Lebensqualität haben. 11. Es ist akzeptabel, Tieren große Schmerzen zuzufügen, wenn dies notwendig ist, um ein wichtiges menschliches Ziel zu erreichen, z.B. in der medizinischen Forschung. 12. Tieren erhebliche Schmerzen zuzufügen, ist gerechtfertigt, wenn der Zweck ausreichend wichtig ist - z. B. in der medizinischen Forschung. 13. Tiere Stress auszusetzen und ihr Wohlbefinden zu beeinträchtigen, ist gerechtfertigt, wenn der Zweck ausreichend wichtig ist.
**Teil C**	**K-12 One Health Education Survey der One Health Comission (2024)** **One Health ist ein ganzheitlicher Ansatz, basierend auf der Idee, dass die Gesundheit von Menschen, Tieren und Ökosystemen eng miteinander verbunden und voneinander abhängig ist (WHO, 2024).** Nachfolgend finden Sie einige Aussagen zum One-Health-Ansatz. Geben Sie bitte für jede Aussage an, in welchem Maße Sie mit ihr übereinstimmen. (1 = stimme gar nicht zu, 6 = stimme völlig zu)	1. Die Gesundheit von Menschen, anderen Tierarten und Pflanzen kann nicht getrennt werden. 2. Die “Umwelt” umfasst die natürliche und die gebaute Umwelt. 3. Die Menschen haben eine moralische Verpflichtung, sich den Herausforderungen von One Health zu stellen. 4. One Health sollte so praktiziert werden, dass es zu keinem Nettoverlust der biologischen Vielfalt (des Ökosystems) kommt. 5. One Health erkennt den Wert sozialer Interaktion als entscheidende Komponente von Gesundheit und Wohlbefinden an. 6. Die Weltgesundheitsorganisation definiert “Gesundheit” als “einen Zustand des vollständigen körperlichen, geistigen und sozialen Wohlbefindens und nicht nur das Fehlen von Krankheit oder Gebrechen”. Diese Definition gilt auch für andere Tiere und Ökosysteme. 7. One Health erkennt den intrinsischen Wert des Lebens auf der Erde (Pflanzen, Tiere, Mikroben) an, unabhängig von einem direkten Nutzen für den Menschen. 8. Ökologische, wirtschaftliche, soziale/kulturelle, ethische und juristische Nachhaltigkeit sind für One Health gleichermaßen wichtig. 9. Wenn man die Gesundheit für eine Spezies optimiert, wird die Gesundheit für andere marginalisiert oder eliminiert.
**Teil D** **(Nur in der Post Befragung)**	1. Nachfolgend finden Sie einige Aussagen zur Beziehung zur Relevanz der Mensch-Tier-Beziehung und One Health auf Ihr Studium bzw. Das Studium Anderer im Sinne der generellen Ausrichtung derzeitiger Studiengänge. Geben Sie bitte für jede Aussage an, in welchem Maße Sie mit ihr übereinstimmen. (1 = stimme gar nicht zu, 6 = stimme völlig zu)	• Es ist wichtig, das komplexe Zusammenspiel zwischen Mensch, Tier und Umwelt im Studium zu behandeln. • Ich habe das Gefühl, durch die Ringvorlesung „Mensch-Tier-Beziehung“ein besseres Verständnis für die Relevanz des Themas und interdisziplinäre Zusammenarbeit erlangt zu haben. • Ich habe den Eindruck, dass Studierende (an meiner Universität) ausreichend Bildungsangebote im Bereich Mensch-Tier-Beziehungen und One Health erhalten.
	2. Bitte schreiben Sie, wie die Themen rund um Mensch-Tier-Beziehungen und One Health bestenfalls in das Curriculum Ihres Studiengangs/Ihnen bekannter Studiengänge integriert werden könnten (schreiben Sie so viel oder so wenig wie Sie mögen).	___________________________ (Freitext)
	3. Wir freuen uns über Feedback (Lob und Kritik) zum Kurs „Mensch-Tier-Beziehung“. Teilen Sie dies gerne im Freitext mit.	___________________________ (Freitext)

**Table:** 

Section	Question	Answer options
**Section A: Demographics**	Please indicate your biological sex.	• Female • Male • Non-binary / gender-queer • No gender • Prefer not to say
	Please indicate your age (age in years, e.g., 30).	*_____* years
	How many people live in your household? (1 = I live alone)	_____ (numeric value)
**Section A: Higher education**	What is your highest general school-leaving qualification?	• I left school without a lower secondary school certificate • I completed a lower secondary school certificate • I completed an intermediate secondary school certificate • I completed a Polytechnic Secondary School certificate (10th grade; before 1965: 8th grade) • I completed a university entrance qualification for universities of applied sciences • I completed a general or subject-specific university entrance qualification (Abitur) • I completed another school-leaving qualification, namely: __________________________
	I study at the following university:	• Witten/Herdecke University • Other: _______________
	I am enrolled in the following degree program:	• Human Medicine • Dentistry • Philosophy, Politics and Economics (B.A.) • Management (B.Sc.) • General Management (M.A.) • Psychology / Psychotherapy (B.Sc.) • Clinical Psychology / Psychotherapy (M.Sc.) • Community Health Nursing (M.Sc.) • Ethics and Organization (M.A.) • Nursing Science (M.Sc.) • Other: _____________________
	The degree awarded in my current program is:	• Bachelor () • Master () • State examination () • Other: _________
	I am currently in the following semester:	_____ (numeric value)
**Section A:** **Other information**	Relation to Healthcare:	• I currently work in the healthcare sector. o If yes: For how many years? o If yes: In the following profession: _______ • I have previously worked in the healthcare sector but no longer do so. o If yes: For how many years? o If yes: In the following profession: ________
	Activism:	• I am actively engaged in animal welfare. (1 = strongly disagree, 6 = strongly agree) • I am actively engaged in climate protection, e.g., in a Campus for Future activist group. (1 = strongly disagree, 6 = strongly agree)
	Pets:	• I currently own pets (Yes / No) o If yes, how many pets? o If yes, which pets? • I have had pets in the past but not currently (Yes / No) o If yes, how many pets? o If yes, which pets?
	Livestock	• I currently own livestock (Yes / No) o If yes, how many animals? o If yes, which animals? • I have had livestock in the past but not currently (Yes / No) o If yes, how many animals? o If yes, which animals?
**Section B**	**Animal Ethics Orientation (Lund et al., 2019)** Below you will find several statements about the relationship between humans and animals. Please indicate the extent to which you agree with each statement. (1 = strongly disagree, 6 = strongly agree)	1. The use of animals by humans should be prohibited by law. 2. In principle, the use of animals by humans is unacceptable because animals can feel pain, happiness, etc. 3. In principle, the use of animals by humans is unacceptable because animals are sentient beings. 4. We have the right to use animals because humans are intellectually superior to animals. 5. Human interests are more important than those of animals. 6. We must prioritize humans over animals. 7. It is acceptable for humans to put animals down if it is done painlessly. 8. Using animals for important human purposes (e.g. medical research) is acceptable if it is done so that the animals do not experience unnecessary stress. 9. Using animals for important human purposes is acceptable if it is done so that the animals do not experience unnecessary pain. 10. Using animals for important human purposes is acceptable if the animals have a decent quality of life. 11. Inflicting serious pain on animals is acceptable if it is necessary in order to achieve a vital human goal–e.g. in medical research. 12. Inflicting considerable pain on animals is justified if the purpose is sufficiently important—e.g. medical research. 13. Exposing animals to stress and reducing their welfare is justified if the purpose is sufficiently important.
**Section C**	**K–12 One Health Education Survey of the One Health Commission (2024)** One Health is a holistic approach based on the idea that the health of humans, animals, and ecosystems is closely interconnected and interdependent (WHO, 2024). Below you will find several statements about the One Health approach. Please indicate the extent to which you agree with each statement. (1 = strongly disagree, 6 = strongly agree)	1. The health of humans, other animal species and plants cannot be separated. 2. “Environment” includes natural and built environments. 3. Humans have a moral imperative to address One Health challenges. 4. One Health should be practiced so that there is no net (ecosystem) loss of biological diversity. 5. One Health embraces the value of social interaction as a critical component of health and well-being. 6. The World Health Organization defines “health” as “a state of complete physical, mental and social well-being and not merely the absence of disease or infirmity.” This definition also applies to other animals and ecosystems. 7. One Health recognizes the intrinsic value of life on earth (plants, animals, microbes) regardless of a direct benefit to humans. 8. Ecological, economic, social/cultural, ethical and justicial sustainability are equally important for One Health. 9. When you optimize health for one species, health for others is marginalized or eliminated.
**Section D (Post-survey only)**	1. Below are several statements regarding the relevance of human–animal relationships and One Health for your own studies and for academic programs in general. Please indicate the extent to which you agree with each statement. (1 = strongly disagree, 6 = strongly agree)	• It is important to address the complex interaction between humans, animals, and the environment in academic studies. • I feel that the lecture series “Human–Animal Relationship” has improved my understanding of the relevance of this topic and of interdisciplinary collaboration. • I have the impression that students (at my university) receive sufficient educational opportunities in the field of human–animal relationships and One Health.
	2. Please describe how topics related to human–animal relationships and One Health could best be integrated into the curriculum of your degree program or other programs you are familiar with (write as much or as little as you like).	___________________________ (free text)
	3. We welcome feedback (praise and criticism) on the course “Human–Animal Relationship.” Please share your feedback in the free-text field.	___________________________ (free text)

## Data Availability

The original contributions presented in this study are included in the article. Further inquiries can be directed to the corresponding author.
